# Recent Advances on the Applications of Luminescent Pb^2+^-Containing Metal–Organic Frameworks in White-Light Emission and Sensing

**DOI:** 10.3389/fchem.2021.636431

**Published:** 2021-04-12

**Authors:** Li-Xin Wang, Jing Xiang, Chuan-Hua Li, Chi-Fai Leung, Jing Xiang

**Affiliations:** ^1^ College of Chemistry and Environmental Engineering, Yangtze University, Jingzhou, China; ^2^ Department of Science and Environmental Studies, The Education University of Hong Kong, Hong Kong, China

**Keywords:** metal-organic framework, lead, luminescence, sensor, white-light emitting materials

## Abstract

Luminescent Pb^2+^-based metal–organic frameworks (MOFs) belong to a new class of multifunctional molecular materials with interesting luminescence properties and potential applications within a single crystalline phase. In this mini review, we present the recent advances that have been achieved in their applications as single-phase white-light emitting materials and chemosensors in the last decade. We focus on the trends in the modification of their structures and luminescence by various bridging ligands, and subsequently their multifunctional applications, which may affect the future development of the field.

## Introduction

Metal–organic frameworks (MOFs), a class of coordination crystalline materials involving metal nodes and multi-topic ligands, have attracted broad interest, as a result of their novel structures and various applications (Wang and Astruc, 2019). Until recent years, most of the reported MOFs were constructed on the basis of d-block and f-block metals (Cui et al., 2012; Zhou et al., 2015). In contrast, much less is understood on main-group MOFs, especially Pb^2+^-containing MOFs, mainly as a result of their flexible geometry and nonclassical coordination chemistry. As with other heavier metals, the toxicity of Pb^2+^, a heavy p-block element, has drawn certain environmental concerns. However, the interesting emission properties of Pb^2+^-based materials, which are highly dependent on the coordination environment, and thus their potential for different applications, have also attracted much interest. The optical and electronic properties of Pb^2+^ compounds have recently been explored in various applications, such as Pb^2+^-based perovskite (Nazarenko et al., 2018), white-light emitting material (Peng et al., 2018), X-ray scintillator (Lu et al., 2019), luminescent sensing (Wang et al., 2018), batteries (Hu et al., 2017), nonlinear optical materials (Chen et al., 2020), ferroelectric materials (Gao et al., 2017), and semiconductors (Terpstra et al., 1997). These fascinating properties are closely associated with its heavy atom effect, inert lone pair effect, large ionic radius, and its borderline position on the hard–soft acid–base scale (Chu et al., 2013). Pb^2+^-based MOFs exhibit frequently unique luminescent properties and applications which are seldom realized in other metal-based MOFs, and thus represent an interesting class of functional materials for the study of structure–property correlations. Since the luminescence properties of Pb^2+^-based MOFs are highly important to their functionality, especially in the application as fluorescent sensors, a brief introduction to the nature of their emission properties will be provided initially, which is followed by the discussion on their applications as white-light emitting materials and luminescent sensors.

## Luminescent Properties of Pb^2+^-Based MOFs

Luminescent metal complexes of Ln^3+^, Zn^2+^, Cd^2+^, and Cu^+^, and noble metals (Ru^2+^, Os^2+/6+^, Ir^3+^, Pt^2+^, and Re^+/5+^) have been well documented, and their emission properties are usually predictable (Cui et al., 2012; Zhou et al., 2015). In contrast, the luminescence properties of Pb^2+^ compounds are more complicated, as they may exhibit simultaneously a variety of electronic transitions, including (1) the metal-centered (MC) s→p transition that usually occurs in hemi-directed Pb^2+^ compounds (Pan et al., 2018), (2) ligand-to-metal charge transfer (LMCT) (Pan et al., 2018), (3) Pb^2+^-perturbed ligand-centered π→π* transition (Pan et al., 2018), and (4) metal-to-ligand charge transfer (MLCT) (Chu et al., 2013). The emissions of Pb^2+^ compounds are usually phosphorescence, irrespective of their emission natures, as spin–orbital coupling is enhanced by the heavy atom effect of Pb^2+^. In addition, the luminescence of Pb^2+^-based MOFs is sensitive to the substituents on the ligands and the subtle changes in their macrostructures. Thus, interesting luminescence properties were often reported for Pb^2+^-based MOFs. For example, a novel 3D Pb^2+^ MOF Pb_4_(L^1^)_3_(μ^4^-O)(H_2_O) (1) (H_2_L^1^ = 1,3-benzenedicarboxylate) exhibiting an eight-connected bcu-type topological motif has been synthesized by Yang et al. (2008). Upon excitation at 374 nm, the Pb^2+^ MOF shows an emission at 424 nm, which is assigned to LMCT from delocalized π bonds of carboxylate groups to *p* orbitals of Pb^2+^ ion. In contrast, Pb^2+^-based MOFs with MLCT character are rare, as the Pb(III) state is not readily accessible. However, Sun and coworkers have recently reported two lead(II) carboxyphosphonate compounds [Pb_2_Cl_3_(H_2_L^2^)]·H_2_O (2) and [Pb_2_(HL^2^)(HL^3^)] (3) (H_3_L^2^ = 1-(phosphonomethyl)piperidine-4-carboxylic acid and H_3_L^3^ = 1,3,5-benzenetricarboxylic acid), which show a significant red shift and enhancement of the emission compared with the free H_3_L^3^ ligand, probably attributed to the MLCT transition (Chu et al., 2013). Moreover, owing to the presence of stereochemically active lone pair effect, the emissions from the metal-centered (MC) s→p transition are most commonly found in semidirectionally coordinated Pb^2+^-MOFs.

## Pb^2+^-Based MOFs as Single-Phase White-Light Emitting Materials

In this part, the recent development in Pb^2+^-based organic–inorganic hybrid materials with white-light emission (WLE) and their photophysical properties will be discussed in relation with the structures of the ligands and the MOFs. Materials with WLE have attracted immense interests as a consequence of their potential usage in displays and lightings. Currently, most of the white-light sources are fabricated by a combination of emissions from separate dopants or a blending of multiple components. However, these materials may bring along complications and higher cost in the fabrication process, together with intrinsic problems such as reabsorption, phase separation, and color variation. The construction of single-phase WLE materials is therefore considered an ideal approach to overcome these issues. To achieve high-quality white light, the single-phase materials must exhibit emission with the Commission Internationale de l’Eclairage (CIE) coordinates (0.33, 0.33). Pb^2+^-based MOFs are found to be potential single-phase materials for WLE, since the multiple emitting centers necessary for WLE could be achieved by suitable combination of organic moieties and Pb^2+^, which exhibits emissions of different origins.

Hybrid organic–inorganic lead halide perovskites were reported to be a special class of intrinsic broadband white-light emitters. The incorporation of structurally deformable Pb_m_X_n_ units into MOFs was a convenient method for the crystal engineering of optoelectric materials. In some cases, the emission solely originates from the lead halide units, and the ligands function only as bridging groups to stabilize the MOFs, whereas the introduction of π-conjugated aromatic moiety was suggested to significantly influence the emission properties by their readily accessible and modifiable charge-transfer bands. Several examples of Pb^2+^ halide perovskites bearing aliphatic dicarboxylate linkage groups were reported. For example, two 2D Pb^2+^ halide polymeric complexes [Pb_2_X_2_][L^4^] (X = Cl, 4 and Br, 5) with broadband WLE were obtained from the reactions of PbX_2_ and trans-1,4-cyclohexanedicarboxylic acid (H_2_L^4^) under hydrothermal conditions. These materials were chemically robust over a wide pH range (3–9) and exhibited undiminished luminescence upon UV excitation for 30 days. The WLE was suggested, by DFT calculations, to originate from the Pb–Pb dimerization and Cl–Cl pairing in the [Pb_2_X_2_]^2+^ (X = Cl/Br) layers (Supplementary Table S1) (Yin J. et al., 2019). Two cationic porous 3D organic–metal halide frameworks [Pb_2_Br_2_][L^5^] (6) and [Pb_3_Br_4_][L^6^] (7) were prepared from bromoplumbate and aliphatic dicarboxylate bridging ligands. These compounds exhibit high chemical resistance and intrinsic white-light emission (λ_ex_ = 360 nm) at high quantum efficiency. The WLE spanned the whole visible-light spectrum and was suggested to arise from the electron–phonon coupling in the strongly deformable and anharmonic lattice (Peng et al., 2018). A series of cationic layered lead halide materials, formulated as [Pb_2_X_2_]^2+^[L^5^] (X = F, Cl and Br) (8–10), were later reported by the same group to exhibit intense broadband WLE in the bulk form at an external quantum efficiency up to 11.8% (Zhuang et al., 2017).

**FIGURE 1:**
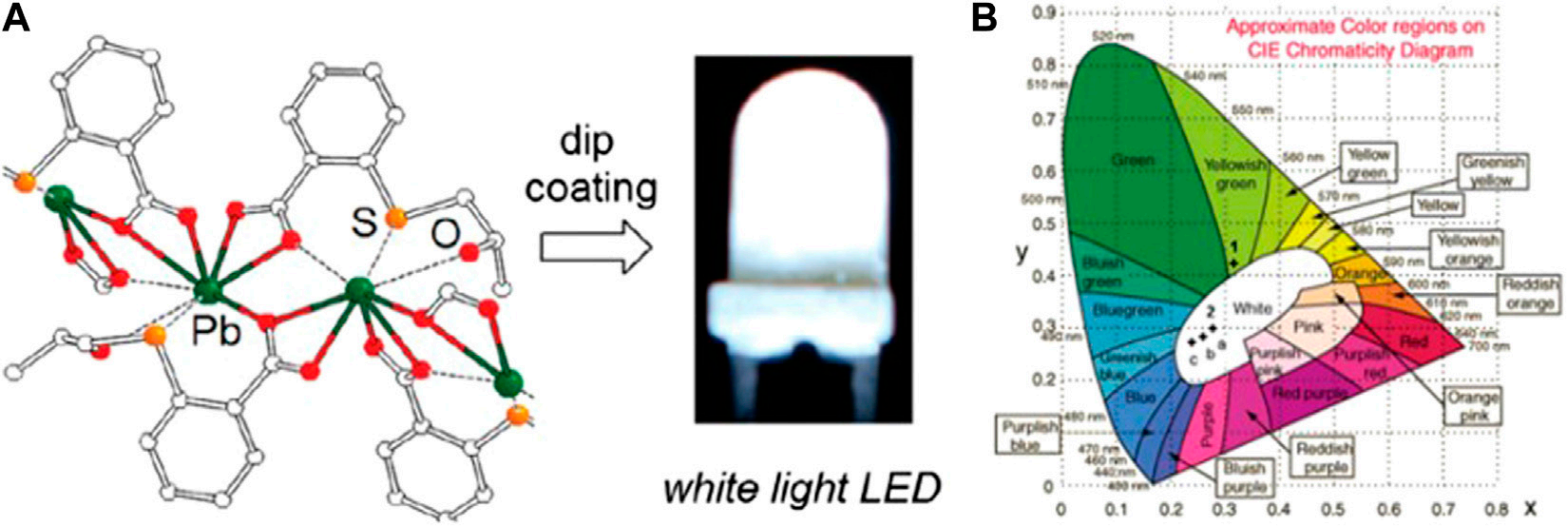
**(A)** Local coordination environment around the Pb^2+^ ion and white LED based on complex [Pb(L^10^)]. **(B)** CIE coordinates for emissions of [Pb(L^9^)] (λ_ex_ = 365 nm) and [Pb(L^10^)].

Aromatic dicarboxylate bridging ligands in lead halide perovskites were found to influence the photophysical properties significantly. Several Pb^2+^ MOFs have been prepared by using derivatized aromatic dicarboxylate bridging moieties, and the study of the dependence of their photophysical properties on the aromatic ring may provide more insights for the development of single-phase WLE materials. These compounds usually exhibit dual or multi-emission bands, in contrast to conventional luminescent materials. Owing to the lone pair effect, the emissions from the MC s→p transition are readily found in Pb^2+^ MOFs with a semi-directional geometry. On the other hand, suitable bridging ligands are crucial in making the ligand-centered and charge-transfer (LMCT/MLCT) transitions accessible in these Pb^2+^ MOFs. Three stable WLE-MOFs, [Pb_2_X_3_^+^][L^7^]_2_ [(CH_3_)_2_NH_2_^+^]_3_ (X = Cl/Br/I) (11–13), were afforded by bridging the deformable [Pb_2_X_3_]^+^ (X = Cl, Br, and I) 1D chains with 1,4-benzenedicarboxylate (H_2_L^7^) (Supplementary Figure S1). Upon near-UV excitation, these materials exhibit intrinsic broadband emissions with a high color-rendering index (CRI) of up to 89 (Peng et al., 2019). Whereas their emissions of 11–13 are mainly originated from the [Pb_2_X_3_^+^] moieties, the introduction of dicarboxylate linkers with rigid aromatic moiety was found to significantly enhance the contribution from the ligand-centered emission and was an effective means for tuning the luminescent properties of the MOFs. For example, single-component broadband photoemitters, [(Pb_4_X_2_)(L^8^)_4_·A_2_]_n_ (X = Cl 14, Br 15, and I 16, A = (CH_3_)_3_NH^+^ and (CH_3_)_2_NH_2_^+^), were formed by bridging 1D haloplumbate chains with the rigid luminescent 2,6-naphthalene dicarboxylate (H_2_L^8^). The bromo and iodo analogs exhibited WLE, which were attributed to the ligand-centered blue emissions from the extended conjugation in L^8^ and the red emissions from the hemi-directed haloplumbate centers (Lin et al., 2020). Xu and coworkers synthesized two emissive 3D networks, PbL^9^ (17) and PbL^10^ (18), with 1,4-benzenedicarboxylic acid modified, respectively, with CH_3_SCH_2_CH_2_S- (L^9^) and (S)-H_3_(OH)CHCH_2_S- (L^10^) at the 2 and 5 positions (Figure 1). The two compounds featured, respectively, a yellowish-green photoluminescence (17) and a bright WLE (18) resulting similarly from broadband dual emissions of different origins. The white emission of 18 was attributed to a suitable ratio of LMCT and s→p transitions, while in 17, the contribution from LMCT was more significant. A thin film of 18 was then applied onto a commercially available UV-LED lamp by a dip-coating procedure (Figure 1) and demonstrated to work in conventional lighting application (He et al., 2012). Wibowo and coworkers have recently synthesized two Pb^2+^-based MOFs, Pb(HL^3^)(1,4-dioxane)0.5 (19) and Pb_2_(HL^3^)_2_(H_2_O)_5_ (20), by using a dissolution–crystallization method (H_3_L^3^ = benzene-1,3,5-tricarboxylic acid). These complexes contained similar linear subunits that were interconnected by HL^3^ into three-dimensional porous MOFs. The two compounds exhibit broad emissions, probably originating from a mixture of ILCT, LMCT, and/or MLCT, upon excitation at λ_ex_ = 350 nm. Particularly, the CIE coordinates (0.33, 0.36) of 20 are close to the ideal CIE coordinates (0.33, 0.33) for WLE (Al-Nubi et al., 2019).

In addition to carboxylate moieties, N-heterocycles have also emerged as important linkage groups in Pb^2+^ MOFs. Zeng and coworkers have recently reported three Pb^2+^ MOFs, [Pb_2_(L^11^)_2_(DMA)]·DMA (21), [Pb_2_(L^11^)_2_(DMF)]·1.5DMF (22), and [Pb_2_(L^11^)_2_(DMF)]·NEt_3_ (23) (H_2_L^11^ = 5-(pyridin-4-yl)isophthalic acid, DMA = *N,N*-dimethylacetamide) (Supplementary Figure S3). These bulk MOF materials had iso-reticular structures with 1D square or rhombic channels with subtle difference in Pb^2+^ coordination geometry. Optical experiments showed that 21 was an excellent white emitter with multiple advantages, including pure white color with the chromaticity coordinates (0.331, 0.347) (λ_ex_ = 350 nm), high fluorescence intensity, and good compatibility to human visibility (Yin Z. et al., 2019). Hydrothermal reaction of pyridine-2,6-dicarboxylic acid (H_2_L^12^) and Pb(NO_3_)_2_ afforded a rhombic-like 2D polymer [Pb(L^12^)] (24) (Qi et al., 2018). Upon excitation at λ_ex_ = 340 nm, 24 exhibits a high-energy emission at 441 nm along with two broad low-energy bands at ca. 553 and 662 nm with a high quantum efficiency of 52%. The emission is possibly assigned to a mixture of LMCT and s→p transition of the Pb^2+^ center. The high quantum yield and thermal stability, as well as CIE coordinate (0.28, 0.25), suggested the compound as a potential candidate for solid-state white luminescent materials. Reaction of the structurally related ligand, pyridine-2,5-dicarboxylate ligand (H_2_L^13^), with Pb(NO_3_)_2_ afforded a 3D [Pb(L^13^)(H_2_O)] (25) (Wibowo et al., 2010), which is formed by connecting the 1D chains of corner-shared distorted capped trigonal prisms with L^13^. 25 is also a single-phase WLE phosphor covering a wide spectral range; however, its luminescence origin remains unclear. Examples of WLE Pb^2+^ MOFs constructed from bridging ligands containing only N-heterocyclic donor moieties are relatively rare but are important for illustrating the influence of aromatic ligands with extended π-conjugation on their emission, because of the more efficient ligand-centered and LMCT transitions. For example, two Pb^2+^-based coordination polymers, [Pb(NO_3_)(L^14^)]_n_ (26) and [Pb(L^14^)_2_]_n_ (27), were synthesized from the reactions of 1-tetrazole-4-imidazole-benzene (HL^14^) and Pb^2+^ salt in different solvents (Chen et al., 2015). Both compounds exhibit dual emission resulting from different emission origins of LMCT and IL π-π* charge transfer. However, their photoluminescence is dependent on the excitation wavelengths. Recently, Peedikakkal and coworkers reported two Pb^2+^ MOFs, [Pb_2_(L^15^)(O_2_CCH_3_)_2_(O_2_CCH_3_)_2_]·H_2_O (28) and [Pb(L^15^)(O_2_CCF_3_)_2_]·1/2CHCl_3_ (29), as well as the mononuclear complex [Pb(L^15^-H)_2_(O_2_CCF_3_)_4_] (30) prepared from the neutral 4,4’-bipyridine (L^15^) (Peedikakkal et al., 2018). The solid-state photoluminescence of 28–30 was investigated at room temperature. Upon photoexcitation (λ_ex_ = 329, 376, and 330 nm, respectively), the compounds showed near-white light emissions with CIE coordinates (0.24, 0.32) for 28, (0.33, 0.39) for 29, and (0.26, 0.31) for 30, which were attributed to mixed LMCT and MC transitions.

## Pb^2+^-Based MOFs as Sensors of Ions and Organic Substrates

MOFs have been regarded as one of the promising candidates of fluorescent probes, as a result of the high sensitivity, short response time, portability, and ease of visualization (Kreno et al., 2012). Recent works on Pb^2+^-based MOFs showed that they exhibited the potential to serve as efficient sensor materials, since their luminescence intensity is found to change linearly with the concentration of the analytes, which are absorbed into the MOF structures. Among the Pb^2+^-based MOFs reported so far, multi-responsive luminescent MOFs which could probe more than one analyte were of particular interest.

As in the abovementioned WLE materials, carboxylates were often adopted as bridging ligands in luminescent Pb^2+^-based MOF sensors of ionic and organic analytes. Typical examples are the Pb^2+^-based MOFs containing pyridine-carboxylates. The addition of various functional groups, such as halides and non-coordinated heteroatoms (N or O) on the pyridyl moiety, were found to significantly alter the functions of Pb^2+^-based MOFs, by varying the interaction with the analytes. Recently, Guo and coworkers synthesized two Pb^2+^ complexes, [Pb(L^16^)_2_]_n_ (31) and [Pb(L^17^)_2_]_n_ (32) (HL^16^ = 5-chloronicotinic acid, and HL^17^ = 5-bromonicotinic acid), to investigate effect of the halo-substituents on the sensing properties. It was revealed that the chloro-containing 31 acted as a multi-response luminescent sensor toward Cr_2_O_7_^2‒^, Fe^3+^, and TNP in DMF solution (Supplementary Table S2) (Guo et al., 2019; Miao, 2019). Upon substituting the halide groups by -NH_2_ and -OH groups, two 3D MOFs, {[Pb_3_(L^18^)_2_Cl_5_]·(H_2_O)}n (33) and [Pb_2_(L^19^)Cl_2_]_n_ (34) (HL^18^ = 5-aminonicotinic acid; H_2_L^19^ = 5-hydroxynicotinic acid), have been synthesized, and their functions as luminescent sensors have been compared. Although the non-coordinated donor groups were expected to strengthen the interactions between the MOFs and analytes, very different activities were observed in the two MOFs. 33 was found to be a heterogeneous catalyst for Knoevenagel condensation reaction and exhibited no sensing properties, while 34 was found to be a luminescence sensor for Fe^3+^ with good recyclability (Zhang et al., 2019). Recently, a 2D framework [PbL^18^(NO_3_)]_n_ (35), obtained from hydrothermal reaction of HL^18^ of Pb(NO_3_)_2_, acted not only as a luminescent sensor for picric acid but also as a temperature sensor (Wang, 2019). More recently, Gai reported a novel Pb^2+^-containing polymer, [Pb(L^20^)]·0.5H_2_O·0.5CH_3_OH (36), containing a zwitterionic ligand 4-carboxy-1-(3,4-dicarboxy-benzyl)-pyridinium chloride (H_3_L^20^Cl) (Supplementary Figure S2). Optical experiments indicated that 36 was a versatile turn-off luminescent sensor, which was multi-responsive toward Cr_2_O_7_^2−^, CrO_4_^2−^, Fe^3+^, and nitrobenzene with a fast response and a high selectivity (Zhao et al., 2020).

As extended π-conjugation in the bridging ligands was suggested to significantly influence the luminescence in Pb^2+^-based MOF, related complexes containing phthalate and its derivatives were also studied for their sensing properties. A pair of enantiomorphic luminescent MOFs, [Pb_10_(L^21^)_7_(NO_3_)_6_(H_2_O)_2_] (37) (1P and 1M) (H_2_L^21^ = 5-methylisophthalic acid), which possess a novel {Pb^18^} wheel and a chiral 3D inorganic connectivity, were reported to act as a rapid and highly selective sensor toward Co_2_^+^ (Han et al., 2014). Modification of the MOF polymeric structures and their sensing properties was also explored by the co-reaction with bridging ligands of other acidic moieties. Dong and coworkers have prepared two luminescent Pb^2+^-phosphonate MOFs, the 2D [Pb_3_(L^22^)_2_(HL^23^)(H_2_O)_2_] (38) and 3D [Pb_2_(L^24^)0.5 (L^25^)(H_2_O)_2_]·H_2_O (39) frameworks, which bore both aromatic carboxylates (H_3_L^23^ = 5-sulfosalicylic acid, and NaH_2_L^25^ = 5-sulfoisophthalic acid sodium) and amino methylenephosphonates (H_2_L^22^ = (morpholinomethyl)phosphonic acid and H_4_L^24^ = (piperazine-1,4-diylbis (methylene))bis (phosphonic acid)) as the bridging ligands, under hydrothermal conditions. The compounds were demonstrated to be highly selective fluorescent probes for sensing thymine and VO_3_^−^, respectively, *via* fluorescent quenching (Supplementary Figure S4) (Cai et al., 2018). Multi-responsive and multifunctional MOFs were also obtained on introduction of N-heterocycles onto the aromatic linkage moieties. The hydrothermal reactions of the 1,4-bis-(imidazol-1-yl)terephthalic acid (H_2_L^26^) with Pb^2+^ in different solvents afforded a 3D framework with two different isomeric forms, [Pb(L^26^)]_n_ (40) ((4,5,6)-*c* net) and [Pb(L^26^)]_n_ (41) (6-c pcu net), which could be used in fluorescent sensing of different ions, that is, Fe^3+^ and Cr_2_O_7_^2−^ (Wang et al., 2018). A new Pb^2+^-based 2D MOF, {[PbNa(L^27^)](H_2_O)(DMF)_2_}_n_ (42) containing the π-conjugated ligand 4’-(1H-tetrazol-5-yl)-[1,10-biphenyl]-3,5-dicarboxylate (H_3_L^27^), was found to not only act as a luminescent sensor for the detection of nitroaromatic compounds and ferric ions but also show excellent activity for the photodegradation of methylene orange (Wu et al., 2018).

**FIGURE 2:**
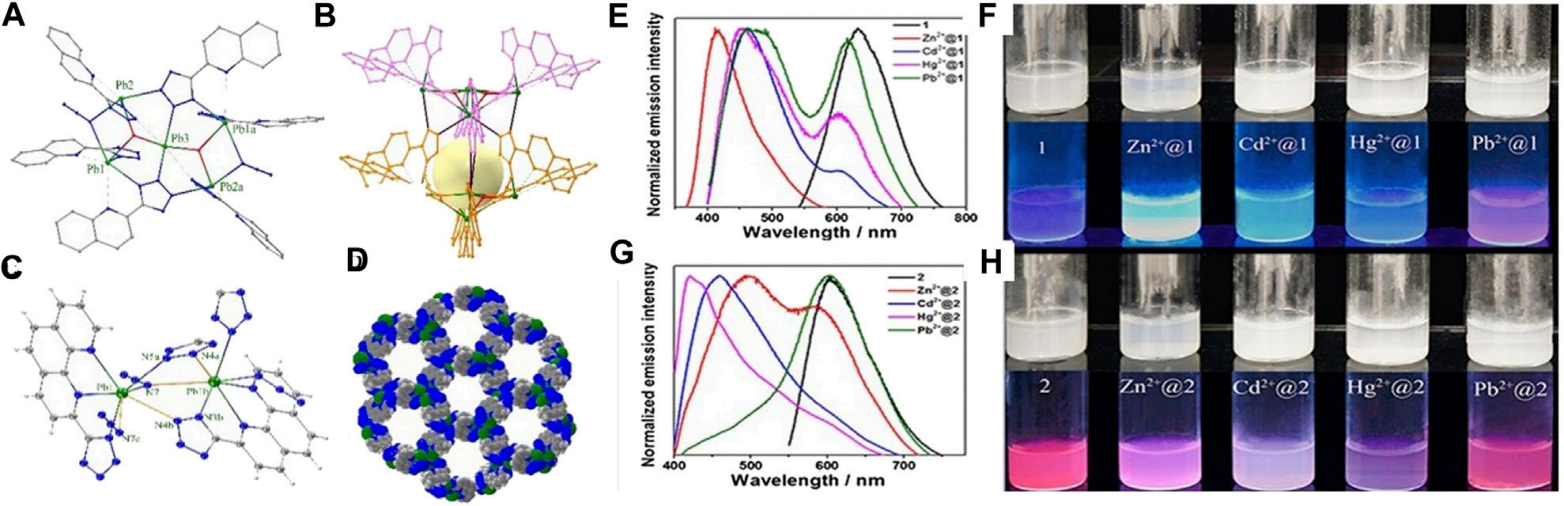
**(A)** Perspective view and **(B)** one of the cavities of 1D chain cage structure of [Pb_5_(L^30^)_6_(N_3_)_2_(OH)_2_] cluster. **(C)** Coordination environment of Pb^2+^ ions in two adjacent molecules and **(D)** 3D porous structure of [Pb(L^31^)(N_3_)]_n_. The emission spectra of **(E)** [Pb_5_(L^30^)_6_(N_3_)_2_(OH)_2_]_n_ and **(G)** [Pb(L_31_)(N_3_)]_n_ suspended in the aqueous solution and in 0.005 M MCl_2_ aqueous solutions (M = Zn^2+^, Cd^2+^, Hg^2+^, and Pb^2+^) with λ_ex_ = 360 nm. The photographs of aqueous suspensions of **(F)** [Pb_5_(L^30^)_6_(N_3_)_2_(OH)_2_]_n_ and **(H)** [Pb(L^31^)(N_3_)]_n_ under normal lighting condition and UV excitation.

Recently, N-heterocycles, for example, imidazolyl and tetrazolyl, with extended π-conjugation, have been adopted as the building blocks in Pb^2+^-based MOF fluorescent probes. As the multiple donor atoms and the π-conjugation on these ligands are sensitive to environmental changes, the resultant MOF fluorescent probes would contain, in their polymeric structures, uncoordinated N-donor atoms which were suggested to interact with different analytes and instruments in multi-responsive luminescent probes for the simultaneous detection of different analytes. The Pb^2+^-based coordination polymer [Pb(L^28^)(NO_3_)_2_]_n_ (43) (L^28^ = 1,4-bis(imidazol-1-yl)benzene), which featured a homochiral double stranded helical structure, was reported to be a luminescent sensor for detecting Fe^3+^ ions in aqueous solution with high sensitivity (Sun et al., 2019). A stable 3D MOF [Pb_3_O_2_L^29^] (44) was obtained from the hydrothermal reaction of 4-(1H-tetrazol-5-yl)phenol ligand (H_2_L^29^) and Pb^2+^ salt. The compound was a sensitive probe for multi-responsive detection of trace amounts of nitroaromatic compounds and Fe^3+^ in aqueous media, with visible color changes (Luo et al., 2017). A cage-containing chain [Pb_5_(L^30^)_6_(N_3_)_2_(OH)_2_]_n_ (45) and an 1D double helical chain [Pb(L^31^)(N_3_)]_n_ (46) with 1D channels were prepared by solvothermal reactions of the tetrazolyl ligands HL^17^ or HL^18^ with Pb^2+^ salts (Figure 2) (HL^30^ = 2-(1H-tetrazol-5-yl)quinoline; HL^31^ = 2-(1H-tetrazol-5-yl)-1,10-phenanthroline) (Xiang et al., 2016). Both MOF structures were reported to uptake different metal ions (Pb^2+^, Hg^2+^, Zn^2+^, and Cd^2+^) and exhibit varied luminescence responses which are transduced as the change in emission wavelengths and are distinguishable with naked eye, rendering them ideal candidates for sensing different heavy metal ions.

## Conclusion and Future Outlook

In this review, we have summarized the development of Pb^2+^-based photoluminescent MOFs. Compared with MOFs of block-d and block-f elements, multifunctional Pb^2+^-based MOFs are still a new area of research. These Pb^2+^ MOFs have shown the potentials for the unique applications, especially in single-phase WLE and ion/molecular sensing, owing to the special coordination features associated with their Pb^2+^ centers. At present, most of Pb^2+^-based MOFs are mainly constructed by bridging carboxylic acid ligands. The design and synthesis of novel functional Pb^2+^-based MOFs containing various N-heterocyclic ligands with multiple donor atoms, such as imidazole and tetrazole, are suggested to be an effective alternative to engineer and tailor their properties for a given purpose. Since these moieties have p*K*_a_ values similar to those of carboxylic acid, their more versatile coordination modes and extended π-conjugated systems will also have a significant influence on the structures and emission properties of the resultant MOFs. In addition, their multiple donor atoms will also provide additional sites of interaction with substrates/analytes of different properties, which may thus result in alternative signal transduction processes and further expand the applications of hybrid organic–inorganic lead halide perovskites and related materials. Considering the toxicity of Pb^2+^ and associated environmental problems, future research will also focus on the synthesis of Pb^2+^-based multifunctional materials with good thermal stability and water stability, to prevent the leakage of Pb^2+^ ion and to realize their practical applications.
