# Supplementary material for: Recent Advances on the Applications of Luminescent Pb2+-Containing Metal–Organic Frameworks in White-Light Emission and Sensing
Source: Front Chem. 2021 Apr 12;9:636431. doi: 10.3389/fchem.2021.636431 (PMC8072004; doi:10.3389/fchem.2021.636431)
Supplement: Supplementary file 1 [file datasheet1.pdf]

Supplementary Material

Supplementary Figures

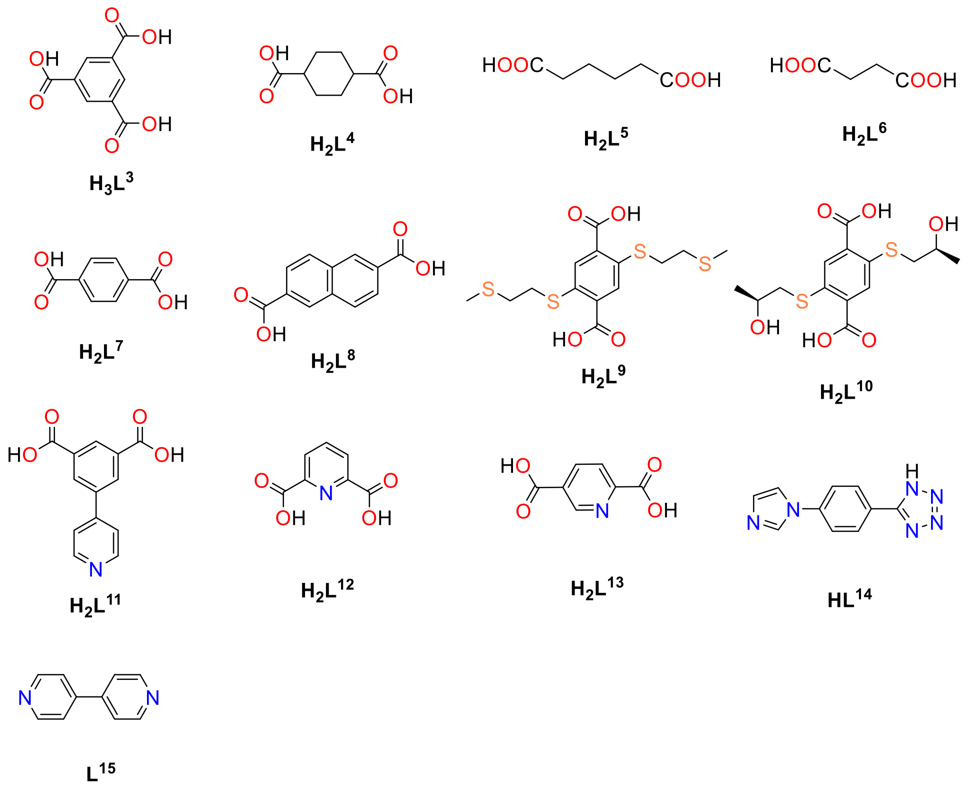

Supplementary Figure 1. The ligands used for WLE materials. (H_3L^3 = benzene-1,3,5tricarboxylic acid; H_2L^4 = trans-1,4-cyclohexanedicarboxylic acid; H_2L^5 = adipic acid; H_2L^6 = succinic acid; H_2L^7=1,4-benzenedicarboxylic acid; H_2L^8 = 2,6-naphthalene dicarboxylic acid; H_2L^9 = 2,5-bis((2-(methylthio)ethyl)thio)terephthalic acid; H_2L^{10} =2,5-bis(((S)-2-hydroxypropyl)thio)terephthalic acid; H_2L^{11} = 5-(pyridin-4-yl)isophthalic acid; H_2L^{12} = pyridine-2,6-dicarboxylic acid); H_2L^{13} = pyridine-2,5-dicarboxylic acid;HL^{14} = 1-tetrazole-4-imidazole-benzene; L^{15} = 4,4′-bipyridine.

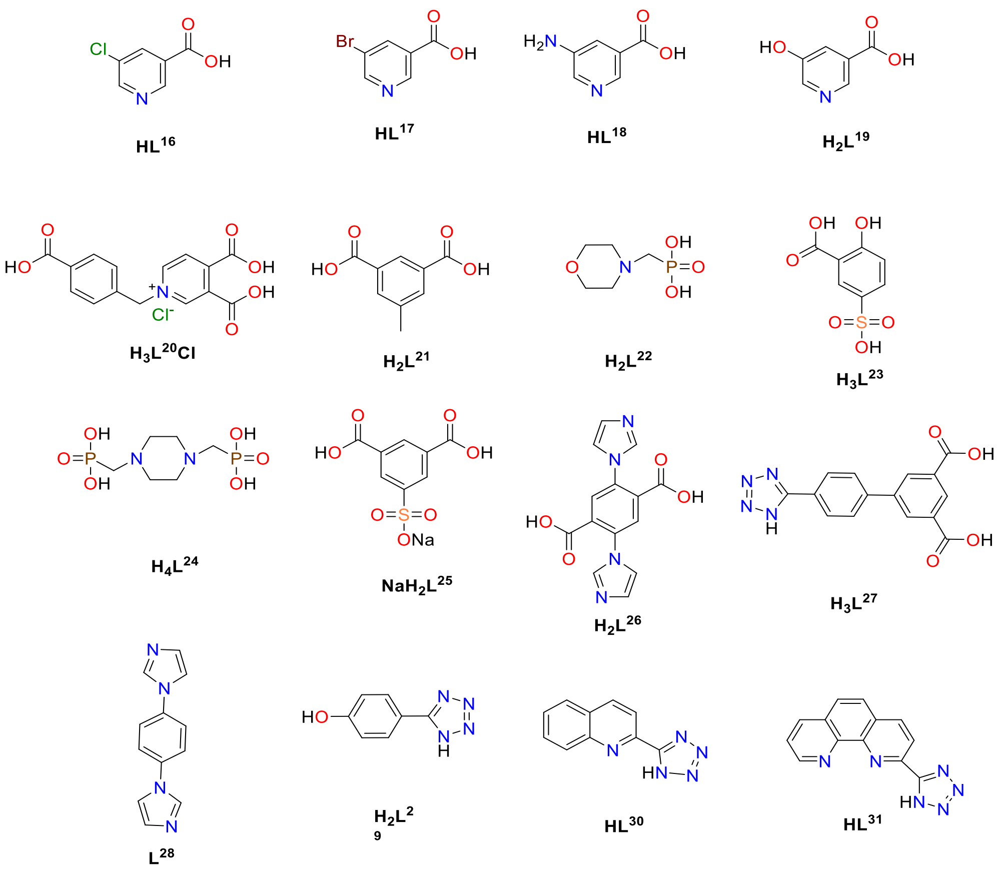

Supplementary Figure 2. The ligands used for Pb^{2+}-based luminescent sensors. (HL^{16} = 5-chloronicotinic acid; HL^{17} = 5-bromonicotinic acid; HL^{18} = 5-aminonicotinic acid; H_2L^{19} = 5-hydroxyni-cotinic acid; $H_3L^{20}Cl$ = 4-carboxy-1-(3,4-dicarboxy-benzyl)-pyridinium chloride; H_2L^{21} = 5-methylisophthalic acid; H_2L^{22} = (morpholinomethyl)phosphonic acid; H_3L^{23} = 5-sulfosalicylic acid; H_4L^{24} = (piperazine-1,4-diylbis(methylene))bis(phosphonic acid); NaH_2L^{25} = 5-sulfoisophthalic acid sodium; H_2L^{26} = 1,4-bis(imidazol-1-yl)terephthalic acid; H_3L^{27} = 4'-(1H-tetrazol-5-yl)-[1,1'-biphenyl]-3,5-dicarboxylic acid; L^{28} = 1,4-bis(imidazol-1-yl)benzene; H_2L^{29} = 4-(1H-tetrazol-5-yl)phenol; HL^{30} = 2-(1*H*-tetrazol-5-yl)quinoline; HL^{31} = 2-(1H-tetrazol-5-yl)-1,10-phenanthroline).

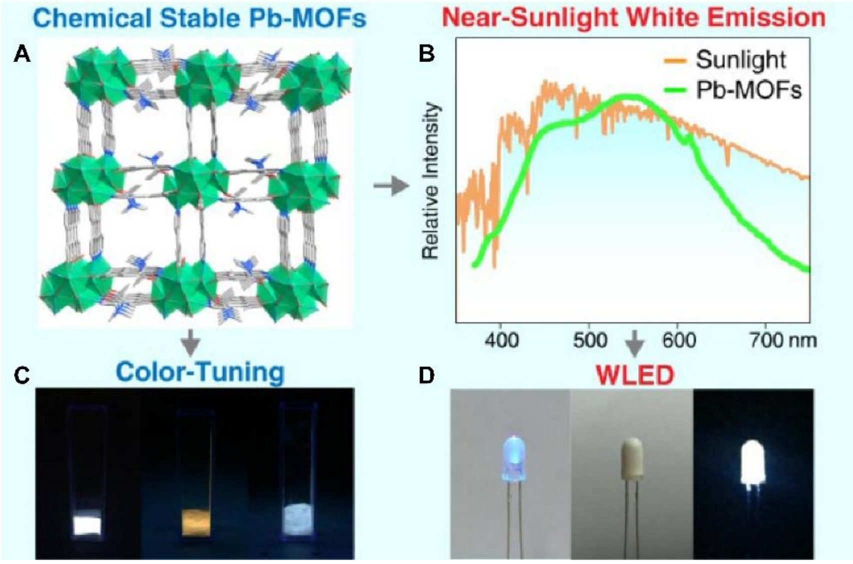

Supplementary Figure 3. (A) View of the rodspacer framework with 1D square channels of $[Pb_2(L^{11})_2(DMA)]\cdot DMA$. **(B)** Comparison of the sunlight spectrum and the emission of $[Pb_2(L^{11})_2(DMA)]\cdot DMA$ under the excitation of 350 nm. **(C)** Photographs of the solid-state emission of $[Pb_2(L^{11})_2(DMA)]\cdot DMA$, $[Pb_2(L^{11})_2(DMF)]\cdot 1.5DMF$ and $[Pb_2(L^{11})_2(DMF)]\cdot NEt_3$ under excitation of 365 nm using an ultraviolet lamp. **(D)** Photographs of a 5 mm UV LED (left, turn-on, λ_{em} = 365 nm), the LED coated with **1** as phosphor (middle), and the WLED emitting white light (right).

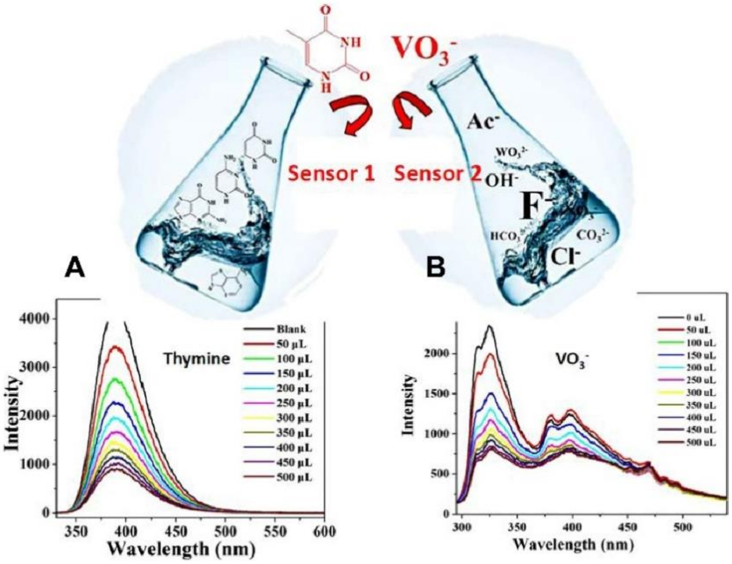

Supplementary Figure 4. (A) The luminescence intensities of suspensions of $[Pb_3[(L^{22})_2(HL^{23})(H_2O)_2]$ in the presence of different amounts (50−500 μL) of thymine aqueous solutions. **(B)** Fluorescence quenching experiments of standard suspensions of $[Pb_2(L^{24})_{0.5}(L^{25})(H_2O)_2]\cdot H_2O$ with the addition of different concentrations of VO_3^-.

Supplementary Table 1. The structural features, emission properties, and proposed emission origins of Pb^{2+}-based WLEs.

Compounds	CNs	Structural features	λ_{em}/nm (λ_{ex}/nm)	Emission origin
$[Pb_2X_2][L^4]$ (X = Cl/Br) (**4，5**)	Cl = 3<br>Br = 4	2D	Cl = 433, 550, 550 (304, 324, 344)<br>Br = 500 (354)	$[Pb_2X_2]^{2+}$
$[Pb_2Br_2][L^5]$ (**6**)	6	3D	530 (360)	Electron-phonon coupling
$[Pb_3Br_4][L^6]$ (**7**)	6	3D	480 (370)	Electron-phonon coupling
$[Pb_2X_2]^{2+}[L^5]$ (X = F, Cl and Br) (**8-10**),	F = 4<br>Cl = 5<br>Br = 5	2D	F = 541 (280)<br>Cl = 536 (327)<br>Br = 565 (356)	Self-trapped excitons and free excitons
$[Pb_2X_3^+][L^7]_2[(CH_3)_2NH_2^+]_3$ (X = Cl/Br/I) (**11-13**)	/	3D	560 (380)	$[Pb_2X_3^+]$
$[(Pb_4X_2)(L^8)_4\cdot A_2]_n$ (X = Cl , Br, and I A=$(CH_3)_3NH^+$ and $(CH_3)_2NH_2^+$) (**14-16**)	Cl = 8<br>Br = 7<br>I = 7	3D	Cl = 388 (340)<br>Br = 393, 684 (374)<br>I = 390, 648 (363)	Cl: LC<br>Br: LC / $[Pb_4X_2]^{6+}$<br>I: LC/ $[Pb_4X_2]^{6+}$
PbL^9 (**17**)	8	3D	468, 531 (365)	LMCT
PbL^{10} (**18**)	8	3D	459, 515	LMCT and s→p transition
$Pb(HL^3)(1,4\text{-dioxane})_{0.5}$ (**19**)	7	3D	530-570 (350)	ILCT and LMCT
$Pb_2(HL^3)_2(H_2O)_5$ (**20**)	9	3D	420-620 (350)	ILCT, LMCT, and/or MLCT
$[Pb_2(L^{11})_2(DMA)]\cdot DMA$ (**21**)	8	1D	460, 545 (350)	LMCT
$[Pb_2(L^{11})_2(DMF)]\cdot 1.5DMF$ (**22**)	8	1D	562 (340)	LMCT
$[Pb_2(L^{11})_2(DMF)]\cdot NEt_3$ (**23**)	8	1D	450, 545 (330, 340, 350)	LMCT
$[Pb(L^{12})]$ (**24**)	5	2D	441, 553, 662 (340)	LMCT/MC
$[Pb(L^{13})(H_2O)]$ (**25**)	7	3D	441, 470, 520 ,563 (386)	LMCT
$[Pb(NO_3)(L^{14})]_n$ (**26**)	5	2D	402, 546 (340)	LMCT and ILCT
$[Pb(L^{14})_2]_n$ (**27**)	5	2D	384,525 (350)	LMCT and ILCT
$[Pb_2(L^{15})(O_2CCH_3)_2(O_2CCH_3)_2]\cdot H_2O$ (**28**)	7	3D	502 (329)	LMCT/ MC
$[Pb(L^{15})(O_2CCF_3)_2]\cdot 1/2CHCl_3$ (**29**)	8-	2D	512, 574 (376)	LMCT/ MC
$[Pb(L^{15}\text{-H})_2(O_2CCF_3)_4]$ (**30**)	6	1D	467 (330)	LMCT/ MC

Supplementary Table 2. The structural features, emission properties, and detecting substrates of Pb^{2+}-based sensors.

Compounds	CNs	Structural features	λ_{em}/nm (λ_{ex}/nm)	Detecting analytes
$[Pb(L^{16})_2]_n$ (**31**)	8	2D	407, 416 (351)	$Cr_2O_7^{2-}$, Fe^{3+}, TNP
$[Pb(L^{17})_2]_n$ (**32)**	6	1D	/	/
$\{[Pb_3(L^{18})_2Cl_5]\cdot(H_2O)\}_n$ (**33**)	8	3D	/	/
$[Pb_2(L^{19})Cl_2]_n$ (**34**)	8	3D	515, 516 (358)	Fe^{3+}
$[PbL^{18}(NO_3)]_n$ (**35**)	7	2D	496 (321)	NACs
$[Pb(L^{20})]\cdot0.5H_2O\cdot0.5CH_3OH$ (**36**)	8	3D	360 (280)	$Cr_2O_7^{2-}$, CrO_4^{2-}, Fe^{3+}, Nitrobenzene
$[Pb_{10}(L^{21})_7(NO_3)_6(H_2O)_2]$ (**37**)	7	3D	565, 603 (396)	Co^{2+}
$[Pb_3[(L^{22})_2(HL^{23})(H_2O)_2]$ (**38**)	8	2D	405, 452 (325)	Thymine molecule
$[Pb_2(L^{24})_{0.5}(L^{25})(H_2O)_2]\cdot H_2O$ (**39**)	8	3D	380, 400 (325)	VO_3^-
$[Pb(L^{26})]_n$ (**40**)	5((4,5,6)-*c* net)	3D	423 (280)	$Cr_2O_7^{2-}$, Fe^{3+}
$[Pb(L^{26})]_n$ (**41**)	6 (6-*c* pcu net)	3D	440 (280)	$Cr_2O_7^{2-}$, Fe^{3+}
$\{[PbNa(L^{27})](H_2O)(DMF)_2\}_n$ (**42**)	6	2D	460 (310)	Nitroaromatic compounds, Fe^{3+}
$[Pb(L^{28})(NO_3)_2]_n$ (**43**)	5	2D	447, 484sh and 525 (304)	Fe^{3+}
$[Pb_3O_2L^{29}]$ (**44**)	4	3D	380 (260)	NACs, Fe^{3+}
$[Pb_5(L^{30})_6(N_3)_2(OH)_2]_n$ (**45**)	4, 8	1D	632 (360)	Zn^{2+}, Cd^{2+}, Hg^{2+}, Pb^{2+}
$[Pb(L^{31})(N_3)]_n$ (**46**)	5	1D	602 (360)	Zn^{2+}, Cd^{2+}, Hg^{2+}, Pb^{2+}
